# Supplementary material for: COVID-19 Vaccination Did Not Increase the Risk of Potentially Related Serious Adverse Events: 18-Month Cohort Study in an Italian Province
Source: Vaccines (Basel). 2022 Dec 23;11(1):31. doi: 10.3390/vaccines11010031 (PMC9861956; doi:10.3390/vaccines11010031)
Supplement: Supplementary file 1 [file vaccines-11-00031-s001.zip › vaccines-2074884-supplementary.pdf]

Table S1. Deaths [A] by COVID-19 vaccine status, infection status, age category, gender and vaccine type.

	Total sample	Unvaccinated	**≥1 Dose** [B]	**1 Dose** [C]	**2 Doses** [D]	**≥3 doses** [E]
Overall sample (N)	(316,315)	(56,494)	(259,821)	(15,832)	(51,684)	(192,305)
% (n)	1.82 (5743)	4.23 (2392)	1.29 (3351)	1.74 (275)	3.52 (1819)	0.65 (1257)
By infection status [F]						
Uninfected (N)	(207,721)	(33,899)	(173,822)	(4015)	(25,243)	(144,564)
% (n)	2.18 (4536)	4.75 (1611)	1.68 (2925)	5.70 (229)	6.65 (1679)	0.70 (1017)
Infected with SARS-CoV-2 (N)	(108,594)	(22,595)	(85,999)	(11,817)	(26,441)	(47,741)
% (n)	1.11 (1207)	3.46 (781)	0.50 (426)	0.39 (46)	0.53 (140)	0.50 (240)
By age						
0-29 years (N)	(76,044)	(18,967)	(57,077)	(5173)	(21,234)	(30,670)
% (n)	0.03 (22)	0.09 (17)	0.01 (5)	0.08 (4)	0.00 (1)	0.00 (0)
30-59 years (N)	(141,563)	(26,097)	(115,466)	(7574)	(21,957)	(85,935)
% (n)	0.26 (374)	0.74 (193)	0.16 (181)	0.40 (30)	0.43 (95)	0.07 (56)
60 or more years (N)	(98,708)	(11,430)	(87,278)	(3085)	(8493)	(75,700)
% (n)	5.42 (5347)	19.09 (2182)	3.63 (3165)	7.81 (241)	20.29 (1723)	1.59 (1201)
By gender						
Females (N)	(161,727)	(27,976)	(133,751)	(7758)	(25,705)	(100,288)
% (n)	1.80 (2906)	4.51 (1261)	1.23 (1645)	1.74 (135)	3.56 (914)	0.59 (596)
Males (N)	(154,588)	(28,518)	(126,070)	(8074)	(25,979)	(92,017)
% (n)	1.84 (2837)	3.97 (1131)	1.35 (1706)	1.73 (140)	3.48 (905)	0.72 (661)
By vaccine type						
BNT162b2 (N)		--	(118,037)	(8110)	(36,108)	(73,819)
% (n)		--	1.84 (2168)	2.08 (169)	3.60 (1301)	0.95 (698)
mRNA-1273 (N)		--	(41,646)	(7500)	(11,202)	(22,944)
% (n)		--	1.64 (684)	1.11 (83)	3.86 (432)	0.74 (1.69)
ChAdOx1 nCoV-19 (N)		--	(2235)	(191)	(2043)	(1)
% (n)		--	3.27 (73)	12.0 (23)	2.45 (50)	0.00 (0)
JNJ-78436735 (N)		--	(549)	--	(549)	(0)
% (n)		--	4.37 (24)	--	4.37 (24)	--
NVX-CoV2373 (N)		--	(147)	(31)	(116)	(0)
% (n)		--	0.0 (0)	0.0 (0)	0.0 (0)	--
Mixed (N) [G]		--	(97,207)	--	(1666)	(95,541)
% (n)		--	0.41 (402)	--	0.72 (12)	0.41 (390)

[A] Subjects deceases from the start of follow-up (January 2, 2021 for the unvaccinated; the day of the first dose for the groups "≥1 dose" and "1 Dose"; the day of the second dose for the group "2 Doses"; the day of the third dose for the group "≥3 Doses") up to July 31, 2022. The deaths were extracted from the official demographic database (Italian "Anagrafica") and the hospital admission discharge abstracts (Italian "SDO").
[B] Subjects who received one or more doses of COVID vaccines between January 2, 2021 and July 31, 2022 (end of follow-up).
[C] Subjects who received only one dose of BNT162b2, mRNA-1273, ChAdOx1 nCoV-19 or NVX-CoV2373.
[D] Subjects who received only one dose of JNJ-78436735 vaccine or only two doses of BNT162b2, mRNA-1273, ChAdOx1 nCoV-19 or NVX-CoV2373 vaccines.
[E] Subjects who received three or more doses of either BNT162b2, mRNA-1273, ChAdOx1 nCoV-19 or NVX-CoV2373 vaccines, or two or more doses of vaccines, if one of the administered vaccines was JNJ-78436735.
[F] Subjects who had at least a positive SARS-CoV-2 swab before the outcome.
[G] Subjects who received two or three different vaccines.

Table S2. Potentially vaccine-related serious adverse events [A], by COVID-19 vaccine status, SARS-CoV-2 infection status, age category, gender and vaccine type.

	Total sample	Unvaccinated	**≥1 Dose** [B]	**1 Dose** [C]	**2 Doses** [D]	**≥3 doses** [E]
Overall sample (N)	(316,315)	(56,494)	(259,821)	(15,913)	(52,067)	(191,841)
% (n)	0.66 (2097)	0.97 (549)	0.60 (1548)	0.88 (140)	1.57 (818)	0.31 (590)
By infection status [F]						
Uninfected (N)	(207,721)	(33,899)	(173,822)	(4063)	(25,538)	(144,221)
% (n)	0.72 (1490)	0.87 (296)	0.69 (1194)	2.26 (92)	2.49 (637)	0.32 (465)
Infected (N)	(108,594)	(22,595)	(85,999)	(11,850)	(26,529)	(47,620)
% (n)	0.56 (607)	1.12 (253)	0.41 (354)	0.41 (48)	0.68 (181)	0.26 (125)
By age						
0-29 years (N)	(76,044)	(18,967)	(57,077)	(5173)	(21,237)	(30,667)
% (n)	0.02 (14)	0.04 (7)	0.02 (7)	0.00 (0)	0.03 (6)	0.00 (1)
30-59 years (N)	(141,563)	(26,097)	(115,466)	(7592)	(22,015)	(85,859)
% (n)	0.20 (280)	0.34 (89)	0.17 (191)	0.38 (29)	0.47 (104)	0.07 (58)
60 or more years (N)	(98,708)	(11,430)	(87,278)	(3148)	(8815)	(75,315)
% (n)	1.83 (1803)	3.96 (453)	1.55 (1350)	3.53 (111)	8.03 (708)	0.71 (531)
By gender						
Females (N)	(161,727)	(27,976)	(133,751)	(7778)	(25,841)	(100,132)
% (n)	0.54 (878)	0.92 (258)	0.46 (620)	0.62 (48)	1.29 (333)	0.24 (239)
Males (N)	(154,588)	(28,518)	(126,070)	(8135)	(26,226)	(91,709)
% (n)	0.79 (1219)	1.02 (291)	0.74 (928)	1.13 (92)	1.85 (485)	0.38 (351)
By vaccine type						
BNT162b2 (N)		--	(118,135)	(8159)	(36,404)	(73,572)
% (n)		--	0.82 (969)	1.03 (84)	1.62 (591)	0.40 (294)
mRNA-1273 (N)		--	(41,657)	(7513)	(11,239)	(22,905)
% (n)		--	0.52 (218)	0.39 (29)	1.17 (132)	0.25 (57)
ChAdOx1 nCoV-19 (N)		--	(2304)	(210)	(2093)	(1)
% (n)		--	4.30 (99)	12.4 (26)	3.49 (73)	0.00 (0)
JNJ-78436735 (N)		--	(551)	--	(551)	(0)
% (n)		--	1.63 (9)	--	1.63 (9)	--
NVX-CoV2373 (N)		--	(147)	(31)	(116)	(0)
% (n)		--	0.68 (1)	3.23 (1)	0.00 (0)	--
Mixed (N) [G]		--	(97,027)	--	(1664)	(95,363)
% (n)		--	0.26 (252)	--	0.78 (13)	0.25 (239)

[A] At least one hospital admission for the selected diseases from the start of follow-up (January 2, 2021 for the unvaccinated; the day of the first dose for the groups "≥1 dose" and "1 Dose"; the day of the second dose for the group "2 Doses"; the day of the third dose for the group "≥3 Doses") up to July 31, 2022. The events were extracted from the Italian SDO database, with the following ICD-9-CM codes in any diagnosis field: 410-410.99 (myocardial infarction); 428.21, 428.31, 428.41 (acute heart failure); 433.01, 433.11, 433.21, 433.31, 433.81, 433.91, 434.01, 434.11, 434.91, or 436 (ischemic stroke); 430-432 (hemorrhagic stroke); 441.00-441.9 (aortic aneurysm); 417.1 or 442.0-9 (peripheral artery aneurysm); 427.5 (cardiac arrest); 414.11 or 414.12 (coronary artery dissection); 452 or 453 (deep vein thrombosis); 415.19 (pulmonary embolism); 391.2, 429.0, 422.0, 422.90 or 422.99 (myocarditis); 391.0, 420.0, 420.90, 420.91, 420.99, 423.1 or 423.2 (pericarditis).
[B] Subjects who received one or more doses of COVID vaccines between January 2, 2021 and July 31, 2022 (end of follow-up).
[C] Subjects who received only one dose of BNT162b2, mRNA-1273, ChAdOx1 nCoV-19 or NVX-CoV2373.
[D] Subjects who received only one dose of JNJ-78436735 vaccine or only two doses of BNT162b2, mRNA-1273, ChAdOx1 nCoV-19 or NVX-CoV2373 vaccines.
[E] Subjects who received three or more doses of either BNT162b2, mRNA-1273, ChAdOx1 nCoV-19 or NVX-CoV2373 vaccines, or two or more doses of vaccines, if one of the administered vaccines was JNJ-78436735.
[F] Subjects who had at least a positive SARS-CoV-2 swab before the outcome.
[G] Subjects who received two or three different vaccines.

Table S3. Myocardial infarction [A] by COVID-19 vaccine status, SARS-CoV-2 infection status, age category, gender and vaccine type.

	Total sample	Unvaccinated	≥1 Dose [B]	1 Dose [C]	2 Doses [D]	≥3 doses [E]
Overall sample (N)	(316,315)	(56,494)	(259,821)	(15,862)	(51,816)	(192,143)
% (n)	0.15 (475)	0.12 (67)	0.16 (408)	0.25 (39)	0.38 (198)	0.09 (171)
By infection status [F]						
Uninfected (N)	(207,721)	(33,899)	(173,822)	(4034)	(25,342)	(144,446)
% (n)	0.17 (349)	0.12 (39)	0.18 (310)	0.62 (25)	0.59 (150)	0.09 (135)
Infected (N)	(108,594)	(22,595)	(85,999)	(11,828)	(26,474)	(47,697)
% (n)	0.12 (126)	0.12 (28)	0.11 (98)	0.12 (14)	0.18 (48)	0.08 (36)
By age						
0-29 years (N)	(76,044)	(18,967)	(57,077)	(5173)	(21,235)	(30,669)
% (n)	0.00 (1)	0.00 (0)	0.00 (1)	0.00 (0)	0.00 (1)	0.00 (0)
30-59 years (N)	(141,563)	(26,097)	(115,466)	(7579)	(21,985)	(85,902)
% (n)	0.06 (91)	0.07 (17)	0.06 (74)	0.09 (7)	0.17 (38)	0.03 (29)
60 or more years (N)	(98,708)	(11,430)	(87,278)	(3110)	(8596)	(75,572)
% (n)	0.39 (383)	0.44 (50)	0.38 (333)	1.03 (32)	1.85 (159)	0.19 (142)
By gender						
Females (N)	(161,727)	(27,976)	(133,751)	(7767)	(25,745)	(100,239)
% (n)	0.10 (156)	0.09 (25)	0.10 (131)	0.17 (13)	0.24 (63)	0.05 (55)
Males (N)	(154,588)	(28,518)	(126,070)	(8095)	(26,071)	(91,904)
% (n)	0.21 (319)	0.15 (42)	0.22 (277)	0.32 (26)	0.52 (135)	0.13 (116)
By vaccine type						
BNT162b2 (N)		--	(118,066)	(8127)	(36,205)	(73,734)
% (n)		--	0.19 (229)	0.27 (22)	0.38 (137)	0.09 (70)
mRNA-1273 (N)		--	(41,649)	(7505)	(11,213)	(22,931)
% (n)		--	0.12 (52)	0.12 (9)	0.22 (25)	0.08 (18)
ChAdOx1 nCoV-19 (N)		--	(2265)	(199)	(2065)	(1)
% (n)		--	1.59 (36)	4.02 (8)	1.36 (28)	0.00 (0)
JNJ-78436735 (N)		--	(550)	--	(550)	(0)
% (n)		--	0.55 (3)	--	0.55 (3)	--
NVX-CoV2373 (N)		--	(147)	(31)	(116)	(0)
% (n)		--	0.00 (0)	0.00 (0)	0.00 (0)	--
Mixed (N) [G]		--	(97,144)	--	(1667)	(95,477)
% (n)		--	0.09 (88)	--	0.30 (5)	0.09 (83)

[A] At least one hospital admission for myocardial infarction from the start of follow-up (January 2, 2021 for the unvaccinated; the day of the first dose for the groups "≥1 dose" and "1 Dose"; the day of the second dose for the group "2 Doses"; the day of the third dose for the group "≥3 Doses") up to July 31, 2022. The events were extracted from the Italian SDO database, with the following ICD-9-CM codes in any diagnosis field: 410-410.99.
[B] Subjects who received one or more doses of COVID vaccines between January 2, 2021 and July 31, 2022 (end of follow-up).
[C] Subjects who received only one dose of BNT162b2, mRNA-1273, ChAdOx1 nCoV-19 or NVX-CoV2373.
[D] Subjects who received only one dose of JNJ-78436735 vaccine or only two doses of BNT162b2, mRNA-1273, ChAdOx1 nCoV-19 or NVX-CoV2373 vaccines.
[E] Subjects who received three or more doses of either BNT162b2, mRNA-1273, ChAdOx1 nCoV-19 or NVX-CoV2373 vaccines, or two or more doses of vaccines, if one of the administered vaccines was JNJ-78436735.
[F] Subjects who had at least a positive SARS-CoV-2 swab before the outcome.
[G] Subjects who received two or three different vaccines.

Table S4. Acute heart failure by COVID-19 vaccine status, SARS-CoV-2 infection status, age category, gender and vaccine type.

	Total sample	Unvaccinated	≥1 Dose [B]	1 Dose [C]	2 Doses [D]	≥3 doses [E]
Overall sample (N)	(316,315)	(56,494)	(259,821)	(15,832)	(51,688)	(192,301)
% (n)	0.01 (38)	0.01 (4)	0.01 (34)	0.01 (1)	0.03 (18)	0.01 (15)
By infection status [F]						
Uninfected (N)	(207,721)	(33,899)	(173,822)	(4015)	(25,246)	(144,561)
% (n)	0.02 (35)	0.01 (4)	0.02 (31)	0.02 (1)	0.07 (17)	0.01 (13)
Infected (N)	(108,594)	(22,595)	(85,999)	(11,817)	(26,442)	(47,740)
% (n)	0.00 (3)	0.00 (0)	0.00 (3)	0.00 (0)	0.00 (1)	0.00 (2)
By age						
0-29 years (N)	(76,044)	(18,967)	(57,077)	(5173)	(21,234)	(30,670)
% (n)	0.00 (0)	0.00 (0)	0.00 (0)	0.00 (0)	0.00 (0)	0.00 (0)
30-59 years (N)	(141,563)	(26,097)	(115,466)	(7574)	(21,957)	(85,935)
% (n)	0.00 (0)	0.00 (0)	0.00 (0)	0.00 (0)	0.00 (0)	0.00 (0)
60 or more years (N)	(98,708)	(11,430)	(87,278)	(3085)	(8497)	(75,696)
% (n)	0.04 (38)	0.03 (4)	0.04 (34)	0.03 (1)	0.21 (18)	0.02 (15)
By gender						
Females (N)	(161,727)	(27,976)	(133,751)	(7758)	(25,708)	(100,285)
% (n)	0.02 (26)	0.01 (3)	0.02 (23)	0.01 (1)	0.04 (11)	0.01 (11)
Males (N)	(154,588)	(28,518)	(126,070)	(8074)	(25,980)	(92,016)
% (n)	0.01 (12)	0.00 (1)	0.01 (11)	0.00 (0)	0.03 (7)	0.00 (4)
By vaccine type						
BNT162b2 (N)		--	(118,038)	(8110)	(36,110)	(73,818)
% (n)		--	0.02 (25)	0.01 (1)	0.04 (13)	0.01 (11)
mRNA-1273 (N)		--	(41,646)	(7500)	(11,202)	(22,944)
% (n)		--	0.01 (5)	0.00 (0)	0.02 (2)	0.01 (3)
ChAdOx1 nCoV-19 (N)		--	(2236)	(191)	(2044)	(1)
% (n)		--	0.04 (1)	0.00 (0)	0.05 (1)	0.00 (0)
JNJ-78436735 (N)		--	(549)	--	(549)	(0)
% (n)		--	0.18 (1)	--	0.18 (1)	--
NVX-CoV2373 (N)		--	(147)	(31)	(116)	(0)
% (n)		--	0.00 (0)	0.00 (0)	0.00 (0)	--
Mixed (N)		--	(97,205)	--	(1667)	(95,538)
% (n)		--	0.00 (2)	--	0.06 (1)	0.00 (1)

[A] At least one hospital admission for acute heart failure from the start of follow-up (January 2, 2021 for the unvaccinated; the day of the first dose for the groups "≥1 dose" and "1 Dose"; the day of the second dose for the group "2 Doses"; the day of the third dose for the group "≥3 Doses") up to July 31, 2022. The events were extracted from the Italian SDO database, with the following ICD-9-CM codes in any diagnosis field: 428.21, 428.31, 428.41.
[B] Subjects who received one or more doses of COVID vaccines between January 2, 2021 and July 31, 2022 (end of follow-up).
[C] Subjects who received only one dose of BNT162b2, mRNA-1273, ChAdOx1 nCoV-19 or NVX-CoV2373.
[D] Subjects who received only one dose of JNJ-78436735 vaccine or only two doses of BNT162b2, mRNA-1273, ChAdOx1 nCoV-19 or NVX-CoV2373 vaccines.
[E] Subjects who received three or more doses of either BNT162b2, mRNA-1273, ChAdOx1 nCoV-19 or NVX-CoV2373 vaccines, or two or more doses of vaccines, if one of the administered vaccines was JNJ-78436735.
[F] Subjects who had at least a positive SARS-CoV-2 swab before the outcome.
[G] Subjects who received two or three different vaccines.

Table S5. Cardiac arrest by COVID-19 vaccine status, SARS-CoV-2 infection status, age category, gender and vaccine type.

	Total sample	Unvaccinated	≥1 Dose [B]	1 Dose [C]	2 Doses [D]	≥3 doses [E]
Overall sample (N)	(316,315)	(56,494)	(259,821)	(15,832)	(51,688)	(192,301)
% (n)	0.15 (477)	0.39 (221)	0.10 (256)	0.16 (26)	0.27 (141)	0.05 (89)
By infection status [F]						
Uninfected (N)	(207,721)	(33,899)	(173,822)	(4015)	(25,246)	(144,561)
% (n)	0.15 (317)	0.26 (87)	0.13 (230)	0.52 (21)	0.51 (130)	0.05 (79)
Infected (N)	(108,594)	(22,595)	(85,999)	(11,817)	(26,442)	(47,740)
% (n)	0.15 (160)	0.59 (134)	0.03 (26)	0.04 (5)	0.04 (11)	0.02 (10)
By age						
0-29 years (N)	(76,044)	(18,967)	(57,077)	(5173)	(21,234)	(30,670)
% (n)	0.00 (2)	0.01 (2)	0.00 (0)	0.00 (0)	0.00 (0)	0.00 (0)
30-59 years (N)	(141,563)	(26,097)	(115,466)	(7574)	(21,958)	(85,934)
% (n)	0.03 (48)	0.11 (30)	0.02 (18)	0.05 (4)	0.04 (9)	0.01 (5)
60 or more years (N)	(98,708)	(11,430)	(87,278)	(3085)	(8496)	(75,697)
% (n)	0.43 (427)	1.65 (189)	0.27 (238)	0.71 (22)	1.55 (132)	0.11 (84)
By gender						
Females (N)	(161,727)	(27,976)	(133,751)	(7758)	(25,706)	(100,287)
% (n)	0.13 (207)	0.33 (91)	0.09 (16)	0.14 (11)	0.24 (62)	0.04 (43)
Males (N)	(154,588)	(28,518)	(126,070)	(8074)	(25,982)	(92,014)
% (n)	0.17 (270)	0.46 (130)	0.11 (140)	0.19 (15)	0.30 (79)	0.05 (46)
By vaccine type						
BNT162b2 (N)		--	(118,039)	(8110)	(36,112)	(73,817)
% (n)		--	0.14 (162)	0.17 (14)	0.28 (102)	0.06 (46)
mRNA-1273 (N)		--	(41,646)	(7500)	(11,202)	(22,944)
% (n)		--	0.13 (53)	0.12 (9)	0.30 (34)	0.04 (10)
ChAdOx1 nCoV-19 (N)		--	(2235)	(191)	(2043)	(1)
% (n)		--	0.22 (5)	1.57 (3)	0.10 (2)	0.00 (0)
JNJ-78436735 (N)		--	(549)	--	(549)	(0)
% (n)		--	0.36 (2)	--	0.36 (2)	--
NVX-CoV2373 (N)		--	(147)	(31)	(116)	(0)
% (n)		--	0.00 (0)	0.00 (0)	0.00 (0)	--
Mixed (N)		--	(97,205)	--	(1666)	(95,539)
% (n)		--	0.03 (34)	--	0.06 (1)	0.03 (33)

[A] At least one hospital admission for cardiac arrest from the start of follow-up (January 2, 2021 for the unvaccinated; the day of the first dose for the groups "≥1 dose" and "1 Dose"; the day of the second dose for the group "2 Doses"; the day of the third dose for the group "≥3 Doses") up to July 31, 2022. The events were extracted from the Italian SDO database, with the following ICD-9-CM codes in any diagnosis field: 427.5.
[B] Subjects who received one or more doses of COVID vaccines between January 2, 2021 and July 31, 2022 (end of follow-up).
[C] Subjects who received only one dose of BNT162b2, mRNA-1273, ChAdOx1 nCoV-19 or NVX-CoV2373.
[D] Subjects who received only one dose of JNJ-78436735 vaccine or only two doses of BNT162b2, mRNA-1273, ChAdOx1 nCoV-19 or NVX-CoV2373 vaccines.
[E] Subjects who received three or more doses of either BNT162b2, mRNA-1273, ChAdOx1 nCoV-19 or NVX-CoV2373 vaccines, or two or more doses of vaccines, if one of the administered vaccines was JNJ-78436735.
[F] Subjects who had at least a positive SARS-CoV-2 swab before the outcome.
[G] Subjects who received two or three different vaccines.

Table S6. Ischemic stroke by COVID-19 vaccine status, SARS-CoV-2 infection status, age category, gender and vaccine type.

	Total sample	Unvaccinated	≥1 Dose [B]	1 Dose [C]	2 Doses [D]	≥3 doses [E]
Overall sample (N)	(316,315)	(56,494)	(259,821)	(15,855)	(51,790)	(192,176)
% (n)	0.16 (495)	0.18 (103)	0.15 (392)	0.22 (35)	0.44 (229)	0.07 (128)
By infection status [F]						
Uninfected (N)	(207,721)	(33,899)	(173,822)	(4027)	(25,321)	(144,474)
% (n)	0.17 (354)	0.20 (68)	0.16 (286)	0.52 (21)	0.65 (165)	0.07 (100)
Infected (N)	(108,594)	(22,595)	(85,999)	(11,828)	(26,469)	(47,702)
% (n)	0.13 (141)	0.15 (35)	0.12 (106)	0.12 (14)	0.24 (64)	0.06 (28)
By age						
0-29 years (N)	(76,044)	(18,967)	(57,077)	(5173)	(21,234)	(30,670)
% (n)	0.00 (1)	0.00 (0)	0.00 (1)	0.00 (0)	0.00 (1)	0.00 (0)
30-59 years (N)	(141,563)	(26,097)	(115,466)	(7580)	(21,966)	(85,920)
% (n)	0.04 (52)	0.05 (14)	0.03 (38)	0.12 (9)	0.09 (19)	0.01 (10)
60 or more years (N)	(98,708)	(11,430)	(87,278)	(3102)	(8590)	(75,586)
% (n)	0.45 (442)	0.78 (89)	0.40 (353)	0.84 (26)	2.43 (209)	0.16 (118)
By gender						
Females (N)	(161,727)	(27,976)	(133,751)	(7763)	(25,749)	(100,239)
% (n)	0.14 (232)	0.19 (53)	0.13 (179)	0.13 (10)	0.42 (108)	0.06 (61)
Males (N)	(154,588)	(28,518)	(126,070)	(8092)	(26,041)	(91,937)
% (n)	0.17 (263)	0.18 (50)	0.17 (213)	0.31 (25)	0.46 (121)	0.07 (67)
By vaccine type						
BNT162b2 (N)		--	(118,070)	(8126)	(36,194)	(73,750)
% (n)		--	0.22 (257)	0.28 (23)	0.48 (172)	0.08 (62)
mRNA-1273 (N)		--	(41,651)	(7504)	(11,210)	(22,937)
% (n)		--	0.14 (59)	0.11 (8)	0.32 (36)	0.07 (15)
ChAdOx1 nCoV-19 (N)		--	(2249)	(194)	(2054)	(1)
% (n)		--	0.89 (20)	2.06 (4)	0.78 (16)	0.00 (0)
JNJ-78436735 (N)		--	(550)	--	(550)	(0)
% (n)		--	0.36 (2)	--	0.36 (2)	--
NVX-CoV2373 (N)		--	(147)	(31)	(116)	(0)
% (n)		--	0.00 (0)	0.00 (0)	0.00 (0)	--
Mixed (N)		--	(97,154)	--	(1666)	(95,488)
% (n)		--	0.06 (54)	--	0.18 (3)	0.05 (51)

[A] At least one hospital admission for ischemic stroke from the start of follow-up (January 2, 2021 for the unvaccinated; the day of the first dose for the groups "≥1 dose" and "1 Dose"; the day of the second dose for the group "2 Doses"; the day of the third dose for the group "≥3 Doses") up to July 31, 2022. The events were extracted from the Italian SDO database, with the following ICD-9-CM codes in any diagnosis field: 433.01, 433.11, 433.21, 433.31, 433.81, 433.91, 434.01, 434.11, 434.91, or 436.
[B] Subjects who received one or more doses of COVID vaccines between January 2, 2021 and July 31, 2022 (end of follow-up).
[C] Subjects who received only one dose of BNT162b2, mRNA-1273, ChAdOx1 nCoV-19 or NVX-CoV2373.
[D] Subjects who received only one dose of JNJ-78436735 vaccine or only two doses of BNT162b2, mRNA-1273, ChAdOx1 nCoV-19 or NVX-CoV2373 vaccines.
[E] Subjects who received three or more doses of either BNT162b2, mRNA-1273, ChAdOx1 nCoV-19 or NVX-CoV2373 vaccines, or two or more doses of vaccines, if one of the administered vaccines was JNJ-78436735.
[F] Subjects who had at least a positive SARS-CoV-2 swab before the outcome.
[G] Subjects who received two or three different vaccines.

Table S7. Hemorrhagic stroke by COVID-19 vaccine status, SARS-CoV-2 infection status, age category, gender and vaccine type.

	Total sample	Unvaccinated	≥1 Dose [B]	1 Dose [C]	2 Doses [D]	≥3 doses [E]
Overall sample (N)	(316,315)	(56,494)	(259,821)	(15,840)	(51,733)	(192,248)
% (n)	0.10 (306)	0.13 (75)	0.09 (231)	0.11 (18)	0.23 (120)	0.05 (93)
By infection status [F]						
Uninfected (N)	(207,721)	(33,899)	(173,822)	(4021)	(25,282)	(144,519)
% (n)	0.12 (240)	0.15 (51)	0.11 (189)	0.37 (15)	0.38 (97)	0.05 (77)
Infected (N)	(108,594)	(22,595)	(85,999)	(11,819)	(26,451)	(47,729)
% (n)	0.06 (66)	0.11 (24)	0.05 (42)	0.03 (3)	0.09 (23)	0.03 (16)
By age						
0-29 years (N)	(76,044)	(18,967)	(57,077)	(5173)	(21,234)	(30,670)
% (n)	0.01 (6)	0.02 (4)	0.00 (2)	0.00 (0)	0.00 (1)	0.00 (1)
30-59 years (N)	(141,563)	(26,097)	(115,466)	(7576)	(21,966)	(85,924)
% (n)	0.03 (38)	0.03 (8)	0.03 (30)	0.03 (2)	0.09 (20)	0.01 (8)
60 or more years (N)	(98,708)	(11,430)	(87,278)	(3091)	(8533)	(75,654)
% (n)	0.27 (262)	0.55 (63)	0.23 (199)	0.52 (16)	1.16 (99)	0.11 (84)
By gender						
Females (N)	(161,727)	(27,976)	(133,751)	(7759)	(25,732)	(100,260)
% (n)	0.09 (140)	0.14 (40)	0.07 (100)	0.08 (6)	0.23 (58)	0.04 (36)
Males (N)	(154,588)	(28,518)	(126,070)	(8081)	(26,001)	(91,988)
% (n)	0.11 (166)	0.12 (35)	0.10 (131)	0.15 (12)	0.24 (62)	0.06 (57)
By vaccine type						
BNT162b2 (N)		--	(118,047)	(8116)	(36,144)	(73,787)
% (n)		--	0.13 (157)	0.14 (11)	0.25 (89)	0.08 (57)
mRNA-1273 (N)		--	(41,647)	(7500)	(11,210)	(22,937)
% (n)		--	0.06 (27)	0.01 (1)	0.15 (17)	0.04 (9)
ChAdOx1 nCoV-19 (N)		--	(2244)	(193)	(2050)	(1)
% (n)		--	0.80 (18)	3.11 (6)	0.59 (12)	0.00 (0)
JNJ-78436735 (N)		--	(549)	--	(549)	(0)
% (n)		--	0.18 (1)	--	0.18 (1)	--
NVX-CoV2373 (N)		--	(147)	(31)	(116)	(0)
% (n)		--	0.00 (0)	0.00 (0)	0.00 (0)	--
Mixed (N)		--	(97,187)	--	(1664)	(95,523)
% (n)		--	0.03 (28)	--	0.06 (1)	0.03 (27)

[A] At least one hospital admission for hemorrhagic stroke from the start of follow-up (January 2, 2021 for the unvaccinated; the day of the first dose for the groups "≥1 dose" and "1 Dose"; the day of the second dose for the group "2 Doses"; the day of the third dose for the group "≥3 Doses") up to July 31, 2022. The events were extracted from the Italian SDO database, with the following ICD-9-CM codes in any diagnosis field: 430-432.
[B] Subjects who received one or more doses of COVID vaccines between January 2, 2021 and July 31, 2022 (end of follow-up).
[C] Subjects who received only one dose of BNT162b2, mRNA-1273, ChAdOx1 nCoV-19 or NVX-CoV2373.
[D] Subjects who received only one dose of JNJ-78436735 vaccine or only two doses of BNT162b2, mRNA-1273, ChAdOx1 nCoV-19 or NVX-CoV2373 vaccines.
[E] Subjects who received three or more doses of either BNT162b2, mRNA-1273, ChAdOx1 nCoV-19 or NVX-CoV2373 vaccines, or two or more doses of vaccines, if one of the administered vaccines was JNJ-78436735.
[F] Subjects who had at least a positive SARS-CoV-2 swab before the outcome.
[G] Subjects who received two or three different vaccines.

Table S8. Coronary dissection by COVID-19 vaccine status, SARS-CoV-2 infection status, age category, gender and vaccine type.

	Total sample	Unvaccinated	≥1 Dose [B]	1 Dose [C]	2 Doses [D]	≥3 doses [E]
Overall sample (N)	(316,315)	(56,494)	(259,821)	(15,832)	(51,685)	(192,304)
% (n)	0.00 (6)	0.00 (2)	0.00 (4)	0.00 (0)	0.00 (2)	0.00 (2)
By infection status [F]						
Uninfected (N)	(207,721)	(33,899)	(173,822)	(4015)	(25,243)	(144,564)
% (n)	0.00 (4)	0.01 (2)	0.00 (2)	0.00 (0)	0.00 (0)	0.00 (2)
Infected (N)	(108,594)	(22,595)	(85,999)	(11,817)	(26,442)	(47,740)
% (n)	0.00 (2)	0.00 (0)	0.00 (2)	0.00 (0)	0.01 (2)	0.00 (0)
By age						
0-29 years (N)	(76,044)	(18,967)	(57,077)	(5173)	(21,234)	(30,670)
% (n)	0.00 (0)	0.00 (0)	0.00 (0)	0.00 (0)	0.00 (0)	0.00 (0)
30-59 years (N)	(141,563)	(26,097)	(115,466)	(7574)	(21,957)	(85,935)
% (n)	0.00 (1)	0.00 (1)	0.00 (0)	0.00 (0)	0.00 (0)	0.00 (0)
60 or more years (N)	(98,708)	(11,430)	(87,278)	(3085)	(8494)	(75,699)
% (n)	0.01 (5)	0.01 (1)	0.00 (4)	0.00 (0)	0.02 (2)	0.00 (2)
By gender						
Females (N)	(161,727)	(27,976)	(133,751)	(7758)	(25,706)	(100,287)
% (n)	0.00 (2)	0.00 (1)	0.00 (1)	0.00 (0)	0.00 (1)	0.00 (0)
Males (N)	(154,588)	(28,518)	(126,070)	(8074)	(25,979)	(92,017)
% (n)	0.00 (4)	0.00 (1)	0.00 (3)	0.00 (0)	0.00 (1)	0.00 (2)
By vaccine type						
BNT162b2 (N)		--	(118,037)	(8110)	(36,109)	(73,818)
% (n)		--	0.00 (1)	0.00 (0)	0.00 (1)	0.00 (0)
mRNA-1273 (N)		--	(41,646)	(7500)	(11,202)	(22,944)
% (n)		--	0.00 (0)	0.00 (0)	0.00 (0)	0.00 (0)
ChAdOx1 nCoV-19 (N)		--	(2235)	(191)	(2043)	(1)
% (n)		--	0.04 (1)	0.00 (0)	0.05 (1)	0.00 (0)
JNJ-78436735 (N)		--	(549)	--	(549)	(0)
% (n)		--	0.00 (0)	--	0.00 (0)	--
NVX-CoV2373 (N)		--	(147)	(31)	(116)	(0)
% (n)		--	0.00 (0)	0.00 (0)	0.00 (0)	--
Mixed (N)		--	(97,207)	--	(1666)	(95,541)
% (n)		--	0.00 (2)	--	0.00 (0)	0.00 (2)

[A] At least one hospital admission for coronary artery dissection from the start of follow-up (January 2, 2021 for the unvaccinated; the day of the first dose for the groups "≥1 dose" and "1 Dose"; the day of the second dose for the group "2 Doses"; the day of the third dose for the group "≥3 Doses") up to July 31, 2022. The events were extracted from the Italian SDO database, with the following ICD-9-CM codes in any diagnosis field: 414.11 or 414.12.

[B] Subjects who received one or more doses of COVID vaccines between January 2, 2021 and July 31, 2022 (end of follow-up).

[C] Subjects who received only one dose of BNT162b2, mRNA-1273, ChAdOx1 nCoV-19 or NVX-CoV2373.

[D] Subjects who received only one dose of JNJ-78436735 vaccine or only two doses of BNT162b2, mRNA-1273, ChAdOx1 nCoV-19 or NVX-CoV2373 vaccines.

[E] Subjects who received three or more doses of either BNT162b2, mRNA-1273, ChAdOx1 nCoV-19 or NVX-CoV2373 vaccines, or two or more doses of vaccines, if one of the administered vaccines was JNJ-78436735.

[F] Subjects who had at least a positive SARS-CoV-2 swab before the outcome.

[G] Subjects who received two or three different vaccines.

Table S9. Aortic aneurism by COVID-19 vaccine status, SARS-CoV-2 infection status, age category, gender and vaccine type.

	Total sample	Unvaccinated	≥1 Dose [B]	1 Dose [C]	2 Doses [D]	≥3 doses [E]
Overall sample (N)	(316,315)	(56,494)	(259,821)	(15,846)	(51,741)	(192,234)
% (n)	0.06 (192)	0.04 (21)	0.07 (171)	0.11 (18)	0.16 (81)	0.04 (72)
By infection status [F]						
Uninfected (N)	(207,721)	(33,899)	(173,822)	(4024)	(25,287)	(144,511)
% (n)	0.07 (153)	0.05 (16)	0.08 (137)	0.30 (12)	0.26 (67)	0.04 (58)
Infected (N)	(108,594)	(22,595)	(85,999)	(11,822)	(26,454)	(47,723)
% (n)	0.04 (39)	0.02 (5)	0.04 (34)	0.05 (6)	0.05 (14)	0.03 (14)
By age						
0-29 years (N)	(76,044)	(18,967)	(57,077)	(5173)	(21,234)	(30,670)
% (n)	0.00 (0)	0.00 (0)	0.00 (0)	0.00 (0)	0.00 (0)	0.00 (0)
30-59 years (N)	(141,563)	(26,097)	(115,466)	(7575)	(21,960)	(85,931)
% (n)	0.01 (8)	0.00 (1)	0.01 (7)	0.01 (1)	0.01 (3)	0.00 (3)
60 or more years (N)	(98,708)	(11,430)	(87,278)	(3098)	(8547)	(75,633)
% (n)	0.19 (184)	0.17 (20)	0.19 (164)	0.55 (17)	0.91 (78)	0.09 (69)
By gender						
Females (N)	(161,727)	(27,976)	(133,751)	(7761)	(25,712)	(100,278)
% (n)	0.02 (40)	0.03 (7)	0.02 (33)	0.06 (5)	0.04 (11)	0.02 (17)
Males (N)	(154,588)	(28,518)	(126,070)	(8085)	(26,029)	(91,956)
% (n)	0.10 (152)	0.05 (14)	0.11 (138)	0.16 (13)	0.27 (70)	0.06 (55)
By vaccine type						
BNT162b2 (N)		--	(118,052)	(8117)	(36,157)	(73,778)
% (n)		--	0.10 (113)	0.11 (9)	0.18 (65)	0.05 (39)
mRNA-1273 (N)		--	(41,646)	(7503)	(11,208)	(22,935)
% (n)		--	0.05 (20)	0.07 (5)	0.10 (11)	0.02 (4)
ChAdOx1 nCoV-19 (N)		--	(2243)	(195)	(2047)	(1)
% (n)		--	0.40 (9)	2.05 (4)	0.24 (5)	0.00 (0)
JNJ-78436735 (N)		--	(549)	--	(549)	(0)
% (n)		--	0.00 (0)	--	0.00 (0)	--
NVX-CoV2373 (N)		--	(147)	(31)	(116)	(0)
% (n)		--	0.00 (0)	0.00 (0)	0.00 (0)	--
Mixed (N)		--	(97,184)	--	(1664)	(95,520)
% (n)		--	0.03 (29)	--	0.00 (0)	0.03 (29)

[A] At least one hospital admission for aortic aneurysm from the start of follow-up (January 2, 2021 for the unvaccinated; the day of the first dose for the groups "≥1 dose" and "1 Dose"; the day of the second dose for the group "2 Doses"; the day of the third dose for the group "≥3 Doses") up to July 31, 2022. The events were extracted from the Italian SDO database, with the following ICD-9-CM codes in any diagnosis field: 441.00-441.9.
[B] Subjects who received one or more doses of COVID vaccines between January 2, 2021 and July 31, 2022 (end of follow-up).
[C] Subjects who received only one dose of BNT162b2, mRNA-1273, ChAdOx1 nCoV-19 or NVX-CoV2373.
[D] Subjects who received only one dose of JNJ-78436735 vaccine or only two doses of BNT162b2, mRNA-1273, ChAdOx1 nCoV-19 or NVX-CoV2373 vaccines.
[E] Subjects who received three or more doses of either BNT162b2, mRNA-1273, ChAdOx1 nCoV-19 or NVX-CoV2373 vaccines, or two or more doses of vaccines, if one of the administered vaccines was JNJ-78436735.
[F] Subjects who had at least a positive SARS-CoV-2 swab before the outcome.
[G] Subjects who received two or three different vaccines.

Table S10. Peripheral aneurism by COVID-19 vaccine status, SARS-CoV-2 infection status, age category, gender and vaccine type.

	Total sample	Unvaccinated	≥1 Dose [B]	1 Dose [C]	2 Doses [D]	≥3 doses [E]
Overall sample (N)	(316,315)	(56,494)	(259,821)	(15,835)	(51,697)	(192,289)
% (n)	0.01 (47)	0.01 (8)	0.02 (39)	0.02 (3)	0.04 (20)	0.01 (16)
By infection status [F]						
Uninfected (N)	(207,721)	(33,899)	(173,822)	(4015)	(25,258)	(144,549)
% (n)	0.01 (31)	0.01 (4)	0.02 (27)	0.00 (0)	0.07 (17)	0.01 (10)
Infected (N)	(108,594)	(22,595)	(85,999)	(11,820)	(26,439)	(47,740)
% (n)	0.01 (16)	0.02 (4)	0.01 (12)	0.03 (3)	0.01 (3)	0.01 (6)
By age						
0-29 years (N)	(76,044)	(18,967)	(57,077)	(5173)	(21,234)	(30,670)
% (n)	0.00 (0)	0.00 (0)	0.00 (0)	0.00 (0)	0.00 (0)	0.00 (0)
30-59 years (N)	(141,563)	(26,097)	(115,466)	(7575)	(21,958)	(85,933)
% (n)	0.01 (8)	0.01 (2)	0.01 (6)	0.01 (1)	0.01 (2)	0.00 (3)
60 or more years (N)	(98,708)	(11,430)	(87,278)	(3087)	(8505)	(75,686)
% (n)	0.04 (39)	0.05 (6)	0.04 (33)	0.06 (2)	0.21 (18)	0.02 (13)
By gender						
Females (N)	(161,727)	(27,976)	(133,751)	(7758)	(25,709)	(100,284)
% (n)	0.01 (11)	0.01 (3)	0.01 (8)	0.00 (0)	0.02 (6)	0.00 (2)
Males (N)	(154,588)	(28,518)	(126,070)	(8077)	(25,988)	(92,005)
% (n)	0.02 (36)	0.02 (5)	0.02 (31)	0.04 (3)	0.05 (14)	0.02 (14)
By vaccine type						
BNT162b2 (N)		--	(118,041)	(8111)	(36,119)	(73,811)
% (n)		--	0.02 (24)	0.01 (1)	0.04 (14)	0.01 (9)
mRNA-1273 (N)		--	(41,647)	(7501)	(11,204)	(22,942)
% (n)		--	0.01 (5)	0.01 (1)	0.04 (4)	0.00 (0)
ChAdOx1 nCoV-19 (N)		--	(2238)	(192)	(2045)	(1)
% (n)		--	0.13 (3)	0.52 (1)	0.10 (2)	0.00 (0)
JNJ-78436735 (N)		--	(549)	--	(549)	(0)
% (n)		--	0.00 (0)	--	0.00 (0)	--
NVX-CoV2373 (N)		--	(147)	(31)	(116)	(0)
% (n)		--	0.00 (0)	0.00 (0)	0.00 (0)	--
Mixed (N)		--	(97,199)	--	(1664)	(95,535)
% (n)		--	0.01 (7)	--	0.00 (0)	0.01 (7)

[A] At least one hospital admission for peripheral artery aneurysm from the start of follow-up (January 2, 2021 for the unvaccinated; the day of the first dose for the groups "≥1 dose" and "1 Dose"; the day of the second dose for the group "2 Doses"; the day of the third dose for the group "≥3 Doses") up to July 31, 2022. The events were extracted from the Italian SDO database, with the following ICD-9-CM codes in any diagnosis field: 417.1 or 442.0-9.
[B] Subjects who received one or more doses of COVID vaccines between January 2, 2021 and July 31, 2022 (end of follow-up).
[C] Subjects who received only one dose of BNT162b2, mRNA-1273, ChAdOx1 nCoV-19 or NVX-CoV2373.
[D] Subjects who received only one dose of JNJ-78436735 vaccine or only two doses of BNT162b2, mRNA-1273, ChAdOx1 nCoV-19 or NVX-CoV2373 vaccines.
[E] Subjects who received three or more doses of either BNT162b2, mRNA-1273, ChAdOx1 nCoV-19 or NVX-CoV2373 vaccines, or two or more doses of vaccines, if one of the administered vaccines was JNJ-78436735.
[F] Subjects who had at least a positive SARS-CoV-2 swab before the outcome.
[G] Subjects who received two or three different vaccines.

Table S11. Pulmonary embolism by COVID-19 vaccine status, SARS-CoV-2 infection status, age category, gender and vaccine type.

	Total sample	Unvaccinated	≥1 Dose [B]	1 Dose [C]	2 Doses [D]	≥3 doses [E]
Overall sample (N)	(316,315)	(56,494)	(259,821)	(15,835)	(51,711)	(192,275)
% (n)	0.05 (162)	0.10 (56)	0.04 (106)	0.06 (9)	0.12 (63)	0.02 (34)
By infection status [F]						
Uninfected (N)	(207,721)	(33,899)	(173,822)	(4016)	(25,265)	(144,541)
% (n)	0.05 (96)	0.08 (27)	0.04 (69)	0.05 (2)	0.19 (48)	0.01 (19)
Infected (N)	(108,594)	(22,595)	(85,999)	(11,819)	(26,446)	(47,734)
% (n)	0.06 (66)	0.13 (29)	0.04 (37)	0.06 (7)	0.06 (15)	0.03 (15)
By age						
0-29 years (N)	(76,044)	(18,967)	(57,077)	(5173)	(21,234)	(30,670)
% (n)	0.00 (1)	0.01 (1)	0.00 (0)	0.00 (0)	0.00 (0)	0.00 (0)
30-59 years (N)	(141,563)	(26,097)	(115,466)	(7576)	(21,958)	(85,932)
% (n)	0.02 (26)	0.04 (11)	0.01 (15)	0.07 (5)	0.04 (9)	0.00 (1)
60 or more years (N)	(98,708)	(11,430)	(87,278)	(3086)	(8519)	(75,673)
% (n)	0.14 (135)	0.38 (44)	0.10 (91)	0.13 (4)	0.63 (54)	0.04 (33)
By gender						
Females (N)	(161,727)	(27,976)	(133,751)	(7760)	(25,716)	(100,275)
% (n)	0.05 (88)	0.33 (31)	0.04 (57)	0.06 (5)	0.12 (31)	0.02 (21)
Males (N)	(154,588)	(28,518)	(126,070)	(8075)	(25,995)	(92,000)
% (n)	0.05 (74)	0.09 (25)	0.04 (49)	0.05 (4)	0.12 (32)	0.01 (13)
By vaccine type						
BNT162b2 (N)		--	(118,046)	(8111)	(36,131)	(73,804)
% (n)		--	0.06 (67)	0.06 (5)	0.13 (46)	0.02 (16)
mRNA-1273 (N)		--	(41,647)	(7500)	(11,204)	(22,943)
% (n)		--	0.04 (17)	0.00 (0)	0.12 (13)	0.02 (4)
ChAdOx1 nCoV-19 (N)		--	(2239)	(193)	(2045)	(1)
% (n)		--	0.27 (6)	1.55 (3)	0.15 (3)	0.00 (0)
JNJ-78436735 (N)		--	(549)	--	(549)	(0)
% (n)		--	0.18 (1)	--	0.18 (1)	--
NVX-CoV2373 (N)		--	(147)	(31)	(116)	(0)
% (n)		--	0.68 (1)	3.23 (1)	0.00 (0)	--
Mixed (N)		--	(97,193)	--	(1666)	(95,527)
% (n)		--	0.01 (14)	--	0.00 (0)	0.01 (14)

[A] At least one hospital admission for pulmonary embolism from the start of follow-up (January 2, 2021 for the unvaccinated; the day of the first dose for the groups "≥1 dose" and "1 Dose"; the day of the second dose for the group "2 Doses"; the day of the third dose for the group "≥3 Doses") up to July 31, 2022. The events were extracted from the Italian SDO database, with the following ICD-9-CM codes in any diagnosis field: 415.19.
[B] Subjects who received one or more doses of COVID vaccines between January 2, 2021 and July 31, 2022 (end of follow-up).
[C] Subjects who received only one dose of BNT162b2, mRNA-1273, ChAdOx1 nCoV-19 or NVX-CoV2373.
[D] Subjects who received only one dose of JNJ-78436735 vaccine or only two doses of BNT162b2, mRNA-1273, ChAdOx1 nCoV-19 or NVX-CoV2373 vaccines.
[E] Subjects who received three or more doses of either BNT162b2, mRNA-1273, ChAdOx1 nCoV-19 or NVX-CoV2373 vaccines, or two or more doses of vaccines, if one of the administered vaccines was JNJ-78436735.
[F] Subjects who had at least a positive SARS-CoV-2 swab before the outcome.
[G] Subjects who received two or three different vaccines.

Table S12. Deep vein thrombosis by COVID-19 vaccine status, SARS-CoV-2 infection status, age category, gender and vaccine type.

	Total sample	Unvaccinated	≥1 Dose [B]	1 Dose [C]	2 Doses [D]	≥3 doses [E]
Overall sample (N)	(316,315)	(56,494)	(259,821)	(18,539)	(51,697)	(192,285)
% (n)	0.03 (101)	0.05 (30)	0.03 (71)	0.06 (9)	0.07 (38)	0.01 (24)
By infection status [F]						
Uninfected (N)	(207,721)	(33,899)	(173,822)	(4020)	(25,254)	(144,548)
% (n)	0.04 (73)	0.07 (23)	0.03 (50)	0.15 (6)	0.11 (27)	0.01 (17)
Infected (N)	(108,594)	(22,595)	(85,999)	(11,819)	(26,443)	(47,737)
% (n)	0.03 (28)	0.03 (7)	0.02 (21)	0.03 (3)	0.04 (11)	0.01 (7)
By age						
0-29 years (N)	(76,044)	(18,967)	(57,077)	(5173)	(21,234)	(30,670)
% (n)	0.00 (2)	0.00 (0)	0.00 (2)	0.00 (0)	0.01 (2)	0.00 (0)
30-59 years (N)	(141,563)	(26,097)	(115,466)	(7578)	(21,959)	(85,929)
% (n)	0.01 (20)	0.02 (6)	0.01 (14)	0.07 (5)	0.03 (6)	0.00 (3)
60 or more years (N)	(98,708)	(11,430)	(87,278)	(3088)	(8504)	(75,686)
% (n)	0.08 (79)	0.21 (24)	0.06 (55)	0.13 (4)	0.35 (30)	0.03 (21)
By gender						
Females (N)	(161,727)	(27,976)	(133,751)	(7760)	(25,709)	(100,282)
% (n)	0.03 (53)	0.07 (19)	0.03 (34)	0.04 (3)	0.07 (19)	0.01 (12)
Males (N)	(154,588)	(28,518)	(126,070)	(8079)	(25,988)	(92,003)
% (n)	0.03 (48)	0.04 (11)	0.03 (37)	0.07 (6)	0.07 (19)	0.01 (12)
By vaccine type						
BNT162b2 (N)		--	(118,040)	(8113)	(36,120)	(73,807)
% (n)		--	0.04 (43)	0.05 (4)	0.07 (25)	0.02 (14)
mRNA-1273 (N)		--	(41,647)	(7502)	(11,202)	(22,943)
% (n)		--	0.02 (10)	0.03 (2)	0.06 (7)	0.00 (1)
ChAdOx1 nCoV-19 (N)		--	(2239)	(193)	(2045)	(1)
% (n)		--	0.36 (8)	1.55 (3)	0.24 (5)	0.00 (0)
JNJ-78436735 (N)		--	(549)	--	(549)	(0)
% (n)		--	0.00 (0)	--	0.00 (0)	--
NVX-CoV2373 (N)		--	(147)	(31)	(116)	(0)
% (n)		--	0.00 (0)	0.00 (0)	0.00 (0)	--
Mixed (N)		--	(97,199)	--	(1665)	(95,534)
% (n)		--	0.01 (10)	--	0.06 (1)	0.01 (9)

[A] At least one hospital admission for deep vein thrombosis from the start of follow-up (January 2, 2021 for the unvaccinated; the day of the first dose for the groups "≥1 dose" and "1 Dose"; the day of the second dose for the group "2 Doses"; the day of the third dose for the group "≥3 Doses") up to July 31, 2022. The events were extracted from the Italian SDO database, with the following ICD-9-CM codes in any diagnosis field: 452 or 453.

[B] Subjects who received one or more doses of COVID vaccines between January 2, 2021 and July 31, 2022 (end of follow-up).

[C] Subjects who received only one dose of BNT162b2, mRNA-1273, ChAdOx1 nCoV-19 or NVX-CoV2373.

[D] Subjects who received only one dose of JNJ-78436735 vaccine or only two doses of BNT162b2, mRNA-1273, ChAdOx1 nCoV-19 or NVX-CoV2373 vaccines.

[E] Subjects who received three or more doses of either BNT162b2, mRNA-1273, ChAdOx1 nCoV-19 or NVX-CoV2373 vaccines, or two or more doses of vaccines, if one of the administered vaccines was JNJ-78436735.

[F] Subjects who had at least a positive SARS-CoV-2 swab before the outcome.

[G] Subjects who received two or three different vaccines.

Table S13. Myocarditis by COVID-19 vaccine status, SARS-CoV-2 infection status, age category, gender and vaccine type.

	Total sample	Unvaccinated	≥1 Dose [B]	1 Dose [C]	2 Doses [D]	≥3 doses [E]
Overall sample (N)	(316,315)	(56,494)	(259,821)	(15,832)	(51,690)	(192,299)
% (n)	0.00 (9)	0.00 (1)	0.00 (8)	0.00 (0)	0.01 (7)	0.00 (1)
By infection status [F]						
Uninfected (N)	(207,721)	(33,899)	(173,822)	(4015)	(25,248)	(144,559)
% (n)	0.00 (5)	0.00 (0)	0.00 (5)	0.00 (0)	0.02 (5)	0.00 (0)
Infected (N)	(108,594)	(22,595)	(85,999)	(11,817)	(26,442)	(47,740)
% (n)	0.00 (4)	0.00 (1)	0.00 (3)	0.00 (0)	0.01 (2)	0.00 (1)
By age						
0-29 years (N)	(76,044)	(18,967)	(57,077)	(5173)	(21,236)	(30,668)
% (n)	0.00 (3)	0.01 (1)	0.00 (2)	0.00 (0)	0.01 (2)	0.00 (0)
30-59 years (N)	(141,563)	(26,097)	(115,466)	(7574)	(21,960)	(85,932)
% (n)	0.00 (4)	0.00 (0)	0.00 (4)	0.00 (0)	0.02 (4)	0.00 (0)
60 or more years (N)	(98,708)	(11,430)	(87,278)	(3085)	(8494)	(75,699)
% (n)	0.00 (2)	0.00 (0)	0.00 (2)	0.00 (0)	0.01 (1)	0.00 (1)
By gender						
Females (N)	(161,727)	(27,976)	(133,751)	(7758)	(25,706)	(100,287)
% (n)	0.00 (1)	0.00 (0)	0.00 (1)	0.00 (0)	0.00 (1)	0.00 (0)
Males (N)	(154,588)	(28,518)	(126,070)	(8074)	(25,984)	(92,012)
% (n)	0.01 (8)	0.00 (1)	0.01 (7)	0.00 (0)	0.02 (6)	0.00 (1)
By vaccine type						
BNT162b2 (N)		--	(118,038)	(8110)	(36,111)	(73,817)
% (n)		--	0.00 (3)	0.00 (0)	0.01 (3)	0.00 (0)
mRNA-1273 (N)		--	(41,646)	(7500)	(11,204)	(22,942)
% (n)		--	0.00 (2)	0.00 (0)	0.02 (2)	0.00 (0)
ChAdOx1 nCoV-19 (N)		--	(2235)	(191)	(2043)	(1)
% (n)		--	0.00 (0)	0.00 (0)	0.00 (0)	0.00 (0)
JNJ-78436735 (N)		--	(549)	--	(549)	(0)
% (n)		--	0.00 (0)	--	0.00 (0)	--
NVX-CoV2373 (N)		--	(147)	(31)	(116)	(0)
% (n)		--	0.00 (0)	0.00 (0)	0.00 (0)	--
Mixed (N)		--	(97,206)	--	(1667)	(95,539)
% (n)		--	0.00 (3)	--	0.12 (2)	0.00 (1)

[A] At least one hospital admission for myocarditis from the start of follow-up (January 2, 2021 for the unvaccinated; the day of the first dose for the groups "≥1 dose" and "1 Dose"; the day of the second dose for the group "2 Doses"; the day of the third dose for the group "≥3 Doses") up to July 31, 2022. The events were extracted from the Italian SDO database, with the following ICD-9-CM codes in any diagnosis field: 391.2, 429.0, 422.0, 422.90 or 422.99.
[B] Subjects who received one or more doses of COVID vaccines between January 2, 2021 and July 31, 2022 (end of follow-up).
[C] Subjects who received only one dose of BNT162b2, mRNA-1273, ChAdOx1 nCoV-19 or NVX-CoV2373.
[D] Subjects who received only one dose of JNJ-78436735 vaccine or only two doses of BNT162b2, mRNA-1273, ChAdOx1 nCoV-19 or NVX-CoV2373 vaccines.
[E] Subjects who received three or more doses of either BNT162b2, mRNA-1273, ChAdOx1 nCoV-19 or NVX-CoV2373 vaccines, or two or more doses of vaccines, if one of the administered vaccines was JNJ-78436735.
[F] Subjects who had at least a positive SARS-CoV-2 swab before the outcome.
[G] Subjects who received two or three different vaccines.

Table S14. Pericarditis by COVID-19 vaccine status, SARS-CoV-2 infection status, age category, gender and vaccine type.

	Total sample	Unvaccinated	≥1 Dose [B]	1 Dose [C]	2 Doses [D]	≥3 doses [E]
Overall sample (N)	(316,315)	(56,494)	(259,821)	(15,836)	(51,693)	(192,292)
% (n)	0.01 (37)	0.01 (6)	0.01 (31)	0.03 (5)	0.04 (19)	0.00 (7)
By infection status [F]						
Uninfected (N)	(207,721)	(33,899)	(173,822)	(4017)	(25,251)	(144,554)
% (n)	0.01 (25)	0.01 (5)	0.01 (20)	0.07 (3)	0.06 (14)	0.00 (3)
Infected (N)	(108,594)	(22,595)	(85,999)	(11,819)	(26,442)	(47,738)
% (n)	0.01 (12)	0.00 (1)	0.01 (11)	0.02 (2)	0.02 (5)	0.01 (4)
By age						
0-29 years (N)	(76,044)	(18,967)	(57,077)	(5173)	(21,235)	(30,669)
% (n)	0.00 (1)	0.00 (0)	0.00 (1)	0.00 (0)	0.00 (1)	0.00 (0)
30-59 years (N)	(141,563)	(26,097)	(115,466)	(7574)	(21,961)	(85,931)
% (n)	0.00 (7)	0.01 (2)	0.00 (5)	0.00 (0)	0.02 (5)	0.00 (0)
60 or more years (N)	(98,708)	(11,430)	(87,278)	(3089)	(8497)	(75,692)
% (n)	0.03 (29)	0.03 (4)	0.03 (25)	0.16 (5)	0.15 (13)	0.01 (7)
By gender						
Females (N)	(161,727)	(27,976)	(133,751)	(7758)	(25,712)	(100,281)
% (n)	0.01 (15)	0.01 (2)	0.01 (13)	0.01 (1)	0.04 (10)	0.00 (2)
Males (N)	(154,588)	(28,518)	(126,070)	(8078)	(25,981)	(92,011)
% (n)	0.01 (22)	0.01 (4)	0.01 (18)	0.05 (4)	0.03 (9)	0.01 (5)
By vaccine type						
BNT162b2 (N)		--	(118,039)	(8111)	(36,114)	(73,814)
% (n)		--	0.01 (16)	0.02 (2)	0.03 (12)	0.00 (2)
mRNA-1273 (N)		--	(41,646)	(7500)	(11,203)	(22,943)
% (n)		--	0.00 (2)	0.00 (0)	0.01 (1)	0.00 (1)
ChAdOx1 nCoV-19 (N)		--	(2240)	(194)	(2045)	(1)
% (n)		--	0.27 (6)	1.55 (3)	0.15 (3)	0.00 (0)
JNJ-78436735 (N)		--	(549)	--	(549)	(0)
% (n)		--	0.00 (0)	--	0.00 (0)	--
NVX-CoV2373 (N)		--	(147)	(31)	(116)	(0)
% (n)		--	0.00 (0)	0.00 (0)	0.00 (0)	--
Mixed (N)		--	(97,200)	--	(1666)	(95,534)
% (n)		--	0.01 (7)	--	0.18 (3)	0.00 (4)

[A] At least one hospital admission for pericarditis from the start of follow-up (January 2, 2021 for the unvaccinated; the day of the first dose for the groups "≥1 dose" and "1 Dose"; the day of the second dose for the group "2 Doses"; the day of the third dose for the group "≥3 Doses") up to July 31, 2022. The events were extracted from the Italian SDO database, with the following ICD-9-CM codes in any diagnosis field: 391.0, 420.0, 420.90, 420.91, 420.99, 423.1 or 423.2.
[B] Subjects who received one or more doses of COVID vaccines between January 2, 2021 and July 31, 2022 (end of follow-up).
[C] Subjects who received only one dose of BNT162b2, mRNA-1273, ChAdOx1 nCoV-19 or NVX-CoV2373.
[D] Subjects who received only one dose of JNJ-78436735 vaccine or only two doses of BNT162b2, mRNA-1273, ChAdOx1 nCoV-19 or NVX-CoV2373 vaccines.
[E] Subjects who received three or more doses of either BNT162b2, mRNA-1273, ChAdOx1 nCoV-19 or NVX-CoV2373 vaccines, or two or more doses of vaccines, if one of the administered vaccines was JNJ-78436735.
[F] Subjects who had at least a positive SARS-CoV-2 swab before the outcome.
[G] Subjects who received two or three different vaccines.

Table S15. Main outcomes, by COVID-19 vaccination and infection status before and after vaccination *.

	Total sample	Unvaccinated	1 Dose [I]	2 Doses [II]	≥3 doses [III]	≥1 Dose [VI]
Never infected during the follow-up						
Death, overall (N)	(207,721)	(33,899)	(4015)	(25,243)	(144,564)	(173,822)
% (n)	2.18 (4536)	4.75 (1611)	2.29 (570)	6.65 (1679)	0.70 (1017)	1.68 (2925)
HR (95% CI) **	--	1 (Ref. cat.)	1.44 (1.34-1.55)	1.11 (1.09-1.12)	0.76 (0.75-0.77)	0.46 (0.45-0.48)
PVR-SAE, total (N)	(207,721)	(33,899)	(4063)	(25,538)	(144,221)	(173,822)
% (n)	0.72 (1490)	0.87 (296)	2.26 (92)	2.49 (637)	0.32 (465)	0.69 (1194)
HR (95% CI) **	--	1 (Ref. cat.)	1.82 (1.61-2.05)	1.24 (1.21-1.28)	0.87 (0.85-0.88)	0.72 (0.67-0.76)
Infected before vaccination						
Death, overall (N)	(33,815)	(22,595)*	(9731)	(923)	(566)	(11,220)
% (n)	2.39 (807)	3.46 (781)	0.22 (21)	0.54 (5)	0.00 (0)	0.23 (26)
HR (95% CI) **	--	1 (Ref. cat.)	0.31 (0.25-0.38)	0.68 (0.57-0.81)	0.01 (0.00-0.01)	0.28 (0.23-0.35)
PVR-SAE, total (N)	(33,815)	(22,595)*	(9734)	(921)	(565)	(11,220)
% (n)	0.79 (267)	1.12 (253)	0.09 (9)	0.33 (3)	0.35 (2)	0.12 (14)
HR (95% CI) **	--	1 (Ref. cat.)	0.34 (0.24-0.48)	0.79 (0.63-1.00)	0.84 (0.70-1.00)	0.37 (0.28-0.49)
Infected after vaccination						
Death, overall (N)	(97,374)	(22,595)*	(2086)	(25,518)	(47,175)	(74,779)
% (n)	1.21 (1181)	3.46 (781)	1.20 (25)	0.53 (135)	0.51 (240)	0.53 (400)
HR (95% CI) **	--	1 (Ref. cat.)	0.96 (0.84-1.10)	0.82 (0.80-0.85)	0.79 (0.77-0.80)	0.48 (0.47-0.50)
PVR-SAE, total (N)	(97,374)	(22,595)*	(2116)	(25,608)	(47,055)	(74,779)
% (n)	0.61 (593)	1.12 (253)	1.84 (39)	0.70 (178)	0.26 (123)	0.45 (340)
HR (95% CI) **	--	1 (Ref. cat.)	1.54 (1.37-1.72)	1.02 (0.99-1.06)	0.84 (0.81-0.86)	0.69 (0.65-0.72)

* Subjects were considered infected if they had at least one positive SARS-CoV-2 swab before the outcome.
** Adjusted hazards ratios (95% confidence interval - CI) of death and PVR-SAEs, based on Cox proportional hazards models, adjusted for age, gender, infection status, diabetes, hypertension, cardiovascular or cerebrovascular disease, chronic obstructive pulmonary disease, kidney disease, cancer and outcome occurrence before the start of follow-up.
PVR-SAE: Potentially vaccine-related serious adverse events. At least one hospital admission for the selected diseases from the start of follow-up (January 2, 2021 for the unvaccinated; the day of the first dose for the groups "≥1 dose" and "1 Dose"; the day of the second dose for the group "2 Doses"; the day of the third dose for the group "≥3 Doses") up to July 31, 2022. The deaths were extracted from the official demographic database (Italian "Anagrafica") and the hospital admission discharge abstracts (Italian "SDO"). The events were extracted from the Italian SDO database, please see the text for details. The total number of PVR-SAEs does not equal the sum of individual PVR-SAEs because some subjects had more than one event. I Subjects who received only one dose of BNT162b2, mRNA-1273, ChAdOx1 nCoV-19 or NVX-CoV2373. II Subjects who received only one dose of JNJ-78436735 vaccine or only two doses of BNT162b2, mRNA-1273, ChAdOx1 nCoV-19 or NVX-CoV2373 vaccines. III Subjects who received three or more doses of either BNT162b2, mRNA-1273, ChAdOx1 nCoV-19 or NVX-CoV2373 vaccines, or two or more doses of vaccines, if one of the administered vaccines was JNJ-78436735. IV Subjects who received one or more doses of COVID-19 vaccines between January 2, 2021 and July 31, 2022 (end of follow-up).
